# Supplementary material for: Extended fisheries recovery timelines in a changing environment
Source: Nat Commun. 2017 May 19;8:15325. doi: 10.1038/ncomms15325 (PMC5493592; doi:10.1038/ncomms15325)
Supplement: Supplementary Information — Supplementary Figures, Supplementary Table and Supplementary References [file ncomms15325-s1.pdf]

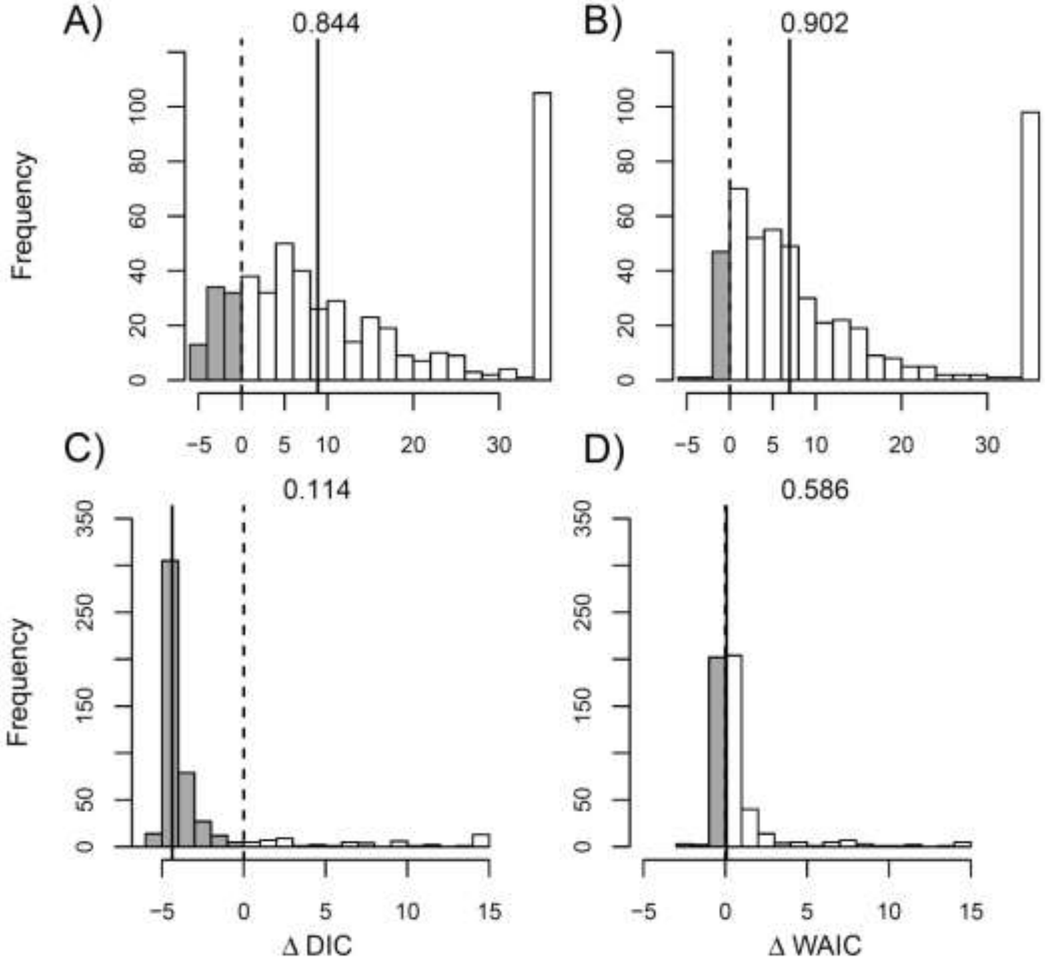

Supplementary Figure 1. Model selection simulation results based on time-varying Graham-Schaefer models with stochastic random walk parameters. Top panels (A, B) represent model selection results for models with time-varying r_t, while bottom panels (C,D) represent models with time-varying K_t. The model specification for K_t is exactly analogous to that given in the Methods of the main text for r_t. Simultaneously time-varying r_t and K_t was not included in the formal estimation due to inherent statistical non-indentifiability. Two model selection criteria were applied: Left panels represent model selection results using the difference in the deviance information criterion[1] (ΔDIC) between fits of the static vs. time-varying model under a simulation of true underlying change in the parameter. The right panels show the analogous plots using the Watanabe-Akaike Information Criterion[2] (ΔWAIC). Results represent model selection experiments under an ensemble of simulations (N=1000) where parameters r, K, σ_r^2, σ_K^2, $r_{t=0}$, $K_{t=0}$, and $B_{t=0}$ were sampled from uniform densities across biologically reasonable parameter ranges. The proportion of model selection simulations classified correctly as nonstationary within the Bayesian analysis is given in each plot.

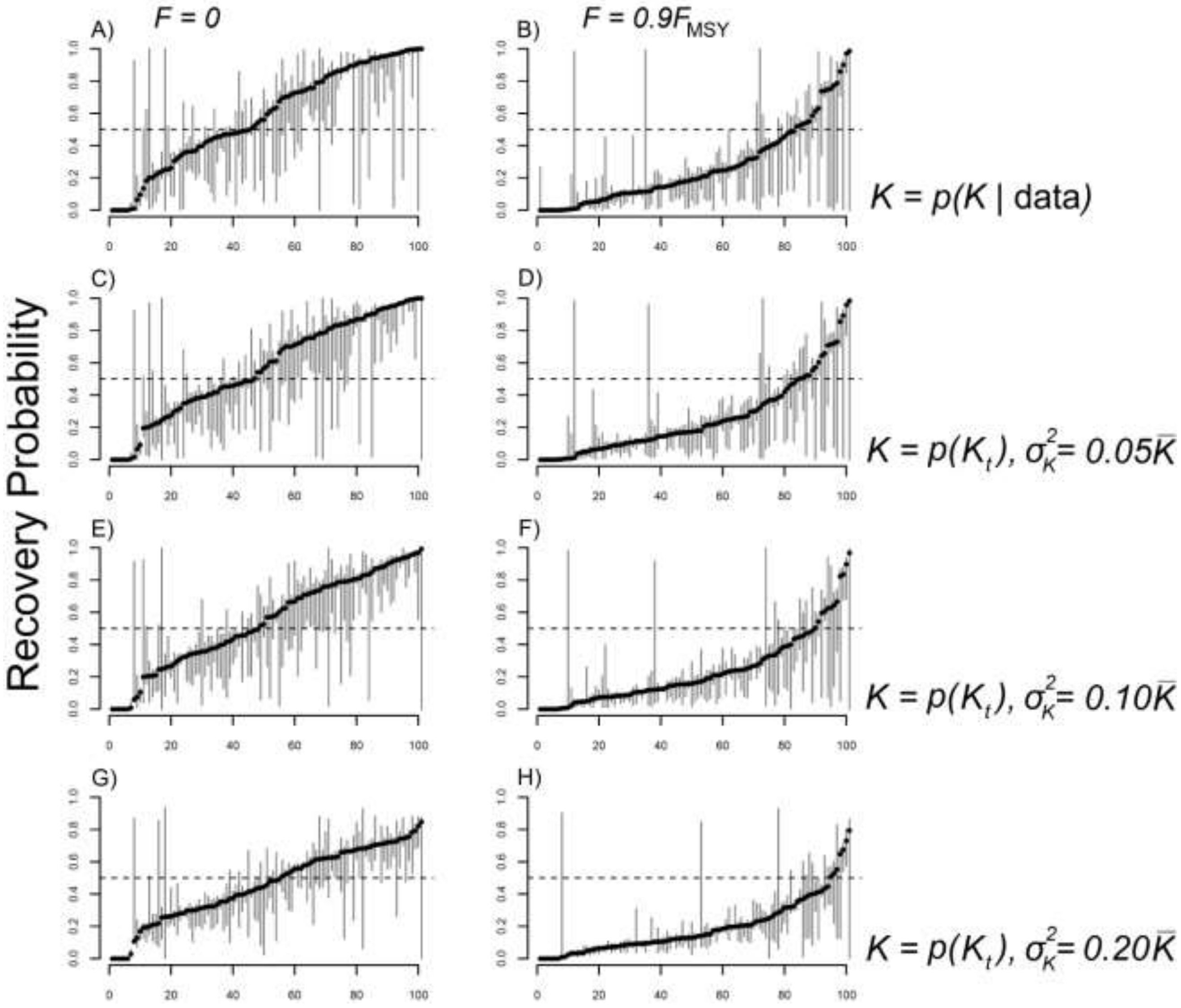

Supplementary Figure 2. Impact of stochastic carrying capacity K_t on forecasted recovery probabilities. Panels A-B are repeated from Fig.4 of the main text where the carrying capacity is assumed static with posterior probability $p(K|\text{data})$. The remaining panels investigate the sensitivity of biomass forecasts to varying degrees of stochastic random walk variation in K_t (and therefore also the recovery target $B_{\text{MSY}} = \frac{1}{2}K_t$). The degree of variation in K_t is set by its random walk variance σ_K^2 where $\overline{K}$ is the mean of $p(K|\text{data})$.

Supplementary Table 1. List of stocks analyzed in the paper. The stock name is given as the 'stocklong' identifier in the RAM Legacy Stock Assessment Database, along with the best mean estimate of stock status (B_0/B_{MSY}).

#	Stock	B_0/B_{MSY}
1	Pacific herring Prince William Sound	0.1
2	Pacific herring Sitka	1.2
3	Alaska plaice Bering Sea and Aleutian Islands	1.75
4	Arrowtooth flounder Bering Sea and Aleutian Islands	2.01
5	Arrowtooth flounder Gulf of Alaska	2.13
6	Atka mackerel Bering Sea and Aleutian Islands	1.47
7	Cabezon Northern California	1.07
8	Cabezon Southern California	0.5
9	Dover sole Gulf of Alaska	1.67
10	Dusky rockfish Gulf of Alaska	2.11
11	Flathead sole Bering Sea and Aleutian Islands	1.8
12	Flathead sole Gulf of Alaska	2.05
13	Greenland turbot Bering Sea and Aleutian Islands	0.32
14	Northern rockfish Bering Sea and Aleutian Islands	2.15
15	Northern rockfish Gulf of Alaska	1.24
16	Northern rock sole Eastern Bering Sea and Aleutian Islands	1.82
17	Pacific cod Bering Sea and Aleutian Islands	1.31
18	Pacific cod Gulf of Alaska	1.32
19	Pacific Ocean perch Eastern Bering Sea and Aleutian Islands	2.09
20	Pacific ocean perch Gulf of Alaska	0.52
21	Rex sole Gulf of Alaska	2
22	Rougheye rockfish Bering Sea and Aleutian Islands	1.67
23	Rougheye rockfish Gulf of Alaska	1.93
24	Red king crab Bristol Bay	1.72
25	Sablefish Eastern Bering Sea / Aleutian Islands / Gulf of Alaska	0.91
26	Snow crab Bering Sea	0.61
27	Shortraker rockfish Bering Sea and Aleutian Islands	1.37
28	Walleye pollock Aleutian Islands	0.32
29	Walleye pollock Eastern Bering Sea	1.19
30	Walleye pollock Gulf of Alaska	0.32
31	Yellowfin sole Bering Sea and Aleutian Islands	1.47
32	Capelin Barents Sea	0.3
33	Atlantic cod coastal Norway	0.63
34	Atlantic cod Northeast Arctic	0.68
35	Greenland halibut Northeast Arctic	0.54
36	Golden Redfish Northeast Arctic	0.7

37	Haddock Northeast Arctic	1.21
38	Pollock Northeast Arctic	1.59
39	Northern shrimp Gulf of Maine	1.11
40	Antarctic toothfish Ross Sea	2.03
41	Southern bluefin tuna Southern Oceans	0.2
42	Anchovy kilka Caspian Sea	0.64
43	Bight redfish Southeast Australia	1.77
44	Deepwater flathead Southeast Australia	1.47
45	common gemfish Southeast Australia	0.16
46	Jackass morwong Southeast Australia	0.38
47	New Zealand ling Eastern half of Southeast Australia	0.87
48	New Zealand ling Western half of Southeast Australia	1.25
49	Orange roughy Cascade Plateau	1.88
50	Orange roughy Southeast Australia	0.8
51	Patagonian toothfish Macquarie Island	1.45
52	Silverfish Southeast Australia	1.37
53	School whiting Southeast Australia	1.13
54	Tiger flathead Southeast Australia	1.24
55	Blue Warehou Eastern half of Southeast Australia	0.15
56	Blue Warehou Western half of Southeast Australia	0.47
57	Atlantic cod NAFO 5Zjm	0.55
58	Haddock NAFO-5Zejm	1.21
59	Haddock NAFO-4X5Y	1.16
60	Atlantic cod NAFO 2J3KL inshore	1.36
61	Atlantic cod NAFO 3Ps	0.8
62	English sole Hecate Strait	1.12
63	Pacific herring Central Coast	0.4
64	Pacific herring Prince Rupert District	0.36
65	Pacific herring Queen Charlotte Islands	0.08
66	Pacific herring Straight of Georgia	1.05
67	Pacific herring West Coast of Vancouver Island	0.2
68	Pacific cod Hecate Strait	0.21
69	Pacific cod West Coast of Vancouver Island	0.25
70	Rock sole Hecate Strait	1.28
71	Pollock NAFO-4X5YZ	0.65
72	Atlantic cod NAFO 3Pn4RS	0.15
73	Herring NAFO 4R spring spawners	0.56
74	Atlantic cod NAFO 4TVn	0.26
75	Herring NAFO 4T fall spawners	1.15
76	Herring NAFO 4T spring spawners	0.26
77	Herring ICES 22-24-IIIa	0.8

78	Herring Northern Irish Sea	0.38
79	Herring North Sea	1.24
80	Herring ICES VIa	0.22
81	Herring ICES VIa-VIIb-VIIc	0.51
82	Albacore tuna North Atlantic	0.78
83	Bluefin tuna Eastern Atlantic	1.22
84	Bigeye tuna Atlantic	0.97
85	Skipjack tuna Eastern Atlantic	1.76
86	Skipjack tuna Western Atlantic	1.81
87	Swordfish Mediterranean Sea	1.23
88	Swordfish North Atlantic	1.17
89	Swordfish South Atlantic	1.22
90	Yellowfin tuna Atlantic	0.95
91	Argentine anchoita Northern Argentina	1.13
92	Argentine anchoita Southern Argentina	1.24
93	Argentine hake Northern Argentina	0.37
94	Argentine hake Southern Argentina	0.65
95	Patagonian grenadier Southern Argentina	1.87
96	Southern blue whiting Southern Argentina	0.87
97	Pacific halibut North Pacific	1.14
98	Anchovy South Africa	1.37
99	Shallow-water cape hake South Africa	1.28
100	South African west coast rock lobster South Africa Areas 1-2	0.01
101	South African west coast rock lobster South Africa Areas 3-4	0.07
102	South African west coast rock lobster South Africa Areas 5-6	0.03
103	South African west coast rock lobster South Africa Area 7	0.06
104	South African west coast rock lobster South Africa Area 8	0.14
105	Cape horse mackerel South Africa South coast	1.36
106	Deep-water cape hake South Africa	0.31
107	Kingklip South Africa	1.17
108	Patagonian toothfish South Africa Subantarctic Prince Edward Islands	0.48
109	South African abalone South Africa	0.63
110	Sardine South Africa	1.2
111	Southern spiny lobster South Africa South coast	0.5
112	American Plaice NAFO-3LNO	0.12
113	American Plaice NAFO-3M	0.27
114	Atlantic cod NAFO 3M	0.11
115	Atlantic cod NAFO 3NO	0.03
116	Greenland halibut NAFO 23KLMNO	0.66
117	Redfish species NAFO 3LN	1.99
118	Redfish species NAFO 3M	0.55

119	Yellowtail Flounder NAFO 3LNO	1.59
120	American Plaice NAFO-5YZ	0.46
121	Atlantic Halibut NAFO-5YZ	0.13
122	Bluefish Atlantic Coast	0.72
123	Black sea bass Mid-Atlantic Coast	1.55
124	Atlantic cod Georges Bank	0.23
125	Atlantic cod Gulf of Maine	1.13
126	Haddock NAFO-5Y	0.87
127	Haddock Georges Bank	0.77
128	Northern shortfin squid Northwestern Atlantic Coast	0
129	Monkfish Gulf of Maine / Northern Georges Bank	1.82
130	Monkfish Southern Georges Bank / Mid-Atlantic	1.71
131	Pollock NAFO-5YZ	0
132	Ocean quahog Atlantic Coast	1.54
133	Sea scallop Georges Bank	1.61
134	Sea scallop Mid-Atlantic Coast	1.54
135	Spiny dogfish Atlantic Coast	1.17
136	Summer flounder Mid-Atlantic Coast	1.22
137	Silver hake Gulf of Maine / Northern Georges Bank	0
138	Silver hake Southern Georges Bank / Mid-Atlantic	0
139	Atlantic surfclam Mid-Atlantic Coast	2.11
140	Tilefish Mid-Atlantic Coast	0.67
141	White hake Georges Bank / Gulf of Maine	0.58
142	Windowpane flounder - Gulf of Maine / Georges Bank	0
143	Windowpane Southern New England-Mid Atlantic	0
144	Winter Flounder NAFO-5Z	1.32
145	Winter Flounder Southern New England-Mid Atlantic	0.26
146	Yellowtail flounder Cape Cod / Gulf of Maine	0.93
147	Yellowtail flounder Georges Bank	0.55
148	Yellowtail Flounder Southern New England-Mid Atlantic	0.11
149	Australian salmon New Zealand	1.04
150	Orange roughy New Zealand Mid East Coast	1.48
151	Atlantic menhaden Atlantic	0.48
152	Arrowtooth flounder Pacific Coast	1.7
153	Blackgill rockfish Pacific Coast	1.42
154	Black rockfish Northern Pacific Coast	1.48
155	Black rockfish Southern Pacific Coast	1.62
156	Blue rockfish California	0.92
157	Bocaccio Southern Pacific Coast	0.37
158	Chilipepper Southern Pacific Coast	1.31
159	Cowcod Southern California	0.08

160	Canary rockfish Pacific Coast	0.44
161	Darkblotched rockfish Pacific Coast	0.56
162	English sole Pacific Coast	2.04
163	Kelp greenling Oregon Coast	1.19
164	Longnose skate Pacific Coast	1.74
165	Longspine thornyhead Pacific Coast	1.63
166	Pacific hake Pacific Coast	0.5
167	Pacific ocean perch Pacific Coast	0.54
168	Petrale sole Northern Pacific Coast	0.8
169	Petrale sole Southern Pacific Coast	0.43
170	Sablefish Pacific Coast	0.89
171	Shortspine thornyhead Pacific Coast	1.44
172	Widow rockfish Pacific Coast	0.86
173	Yelloweye rockfish Pacific Coast	0.38
174	Yellowtail rockfish Northern Pacific Coast	1.32
175	Capelin Iceland	0.87
176	Atlantic cod Faroe Plateau	0.64
177	Atlantic cod Iceland	0.54
178	Haddock Faroe Plateau	1.53
179	Haddock Iceland	1.34
180	Pollock Faroe Plateau	1.67
181	Black oreo West end of Chatham Rise	1.38
182	Smooth oreo Chatham Rise	1.59
183	Smooth oreo West end of Chatham Rise	1.07
184	Hoki Eastern New Zealand	0.91
185	Hoki Western New Zealand	0.47
186	New Zealand snapper New Zealand Area 8	0.2
187	Trevally New Zealand Areas TRE 7	1
188	Red rock lobster New Zealand area CRA1	1.16
189	Red rock lobster New Zealand area CRA2	0.59
190	Red rock lobster New Zealand area CRA3	0.14
191	Red rock lobster New Zealand area CRA4	0.81
192	Red rock lobster New Zealand area CRA5	0.47
193	Red rock lobster New Zealand area CRA7	0.4
194	Red rock lobster New Zealand area CRA8	0.36
195	common gemfish New Zealand	0.63
196	New Zealand ling New Zealand Areas LIN 3 and 4	1.44
197	New Zealand ling New Zealand Areas LIN 5 and 6	1.75
198	New Zealand ling New Zealand Area LIN 6b	1.31
199	New Zealand ling New Zealand Area LIN 72	1.41
200	New Zealand ling New Zealand Area LIN 7WC - WCSI	1.76

201	Southern blue whiting Campbell Island Rise	1.11
202	Southern hake Chatham Rise	0.88
203	Southern hake Sub-Antarctic	1.07
204	New Zealand abalone species New Zealand Area PAU 5A	1.23
205	New Zealand abalone species New Zealand Area PAU 5B	1.04
206	New Zealand abalone species New Zealand Area PAU 5D	0.5
207	New Zealand abalone species New Zealand Area PAU 7	0.81
208	American lobster Rhode Island	0.69
209	Tautog Rhode Island	0.61
210	Winter flounder Rhode Island	0.38
211	Bonnethead shark Atlantic	1.16
212	Blacktip shark Atlantic	2.19
213	Blacktip shark Gulf of Mexico	2.02
214	Gag Gulf of Mexico	1.01
215	Greater amberjack Gulf of Mexico	0.42
216	King mackerel Gulf of Mexico	1.92
217	King mackerel Southern Atlantic Coast	1.69
218	Gulf menhaden Gulf of Mexico	1.37
219	Red grouper Gulf of Mexico	1.88
220	Red porgy Southern Atlantic coast	0.48
221	Sandbar shark Atlantic	0.99
222	Snowy grouper Southern Atlantic coast	0.29
223	Spanish mackerel Southern Atlantic Coast	0.6
224	Tilefish Southern Atlantic coast	0.72
225	Vermilion snapper Southern Atlantic coast	0.79
226	Walleye pollock Northern Sea of Okhotsk	1.61
227	Albacore tuna South Pacific Ocean	0.71
228	Bigeye tuna Western Pacific Ocean	0.71
229	Skipjack tuna Central Western Pacific	1.51
230	Striped marlin Southwestern Pacific Ocean	0.38
231	Yellowfin tuna Central Western Pacific	0.69
232	Chilean jack mackerel Chilean EEZ and offshore	0.35
233	Dover sole Pacific Coast	1.23
234	Gopher rockfish Southern Pacific Coast	2.02
235	Pacific sardine Pacific Coast	1.51
236	Shortbelly rockfish Pacific Coast	0.41
237	Starry flounder Northern Pacific Coast	0.55
238	Starry flounder Southern Pacific Coast	0.93
239	Tasmanian giant crab Tasmania	0.81
240	Walleye pollock Western Bering Sea	1.04
241	Atlantic cod Baltic Areas 22 and 24	0.56

242	Atlantic cod Baltic Areas 25-32	0.24
243	Atlantic cod Kattegat	0.17
244	Herring ICES 25-32	0.65
245	Herring ICES 30	1.64
246	Herring ICES 31	0.62
247	Herring Iceland (Summer spawners)	1.46
248	Herring ICES 28	1.57
249	common European sole ICES Kattegat and Skagerrak	1.21
250	Sprat ICES Baltic Areas 22-32	1.19
251	Fourspotted megrim ICES VIIIc-IXa	1.03
252	Hake Northeast Atlantic North	1
253	Megrim ICES VIIIc-IXa	0.66
254	common European sole Bay of Biscay	1.1
255	Mackerel ICES Northeast Atlantic	1.08
256	Whiting Northeast Atlantic	1.49
257	Atlantic cod Irish Sea	0.37
258	Atlantic cod West of Scotland	0.25
259	Haddock Irish Sea	0
260	Haddock West of Scotland	1.02
261	European Plaice Irish Sea	1.2
262	common European sole Irish Sea	0.67
263	Atlantic cod North Sea	0.2
264	Haddock ICES IIIa and North Sea	0.32
265	Haddock Rockall Bank	1.11
266	Norway pout North Sea	0.4
267	Pollock ICES IIIa, VI and North Sea	0.88
268	Sandeel North Sea	0.4
269	common European sole ICES VIId	1.38
270	Whiting ICES IIIa, VIId and North Sea	0.41
271	Haddock ICES VIIb-k	1.51
272	European Plaice ICES VIIf-g	0.42
273	European Plaice ICES VIIe	0.78
274	common European sole Celtic Sea	0.95
275	common European sole Western English Channel	1.12
276	Whiting ICES VIIe-k	0.79

Supplementary References

1. Gelman, A., Carlin, J. B., Stern, H. S. & Rubin, D. B. *Bayesian data analysis*. (Chapman & Hall/CRC, 2004).

2. Vehtari, A. & Gelman, A. WAIC and cross-validation in Stan. *arXiv* 1–15 (2014).
